# Announcement

**Published:** 2015-05-15

## Click It or Ticket Campaign — May 18–31, 2015

Click It or Ticket, a national campaign coordinated annually by the National Highway Traffic Safety Administration to increase the proper use of seat belts, takes place May 18–31, 2015.

Using a seat belt is one of the most effective ways to prevent serious injury or death in the event of a crash. Using lap/shoulder seat belts can reduce the risk of fatal injury by almost 50% (1). In 2013, more than 21,000 passenger vehicle occupants died in motor vehicle crashes in the United State; 49% were unrestrained at the time of the crash (2). An additional 2.4 million nonfatal injuries were treated in emergency departments in 2013 (3). Yet, millions of persons continue to travel unrestrained (4).

During the 2015 Click it or Ticket campaign,* law enforcement agencies across the nation will conduct intensive, high-visibility enforcement of seat belt laws, during both daytime and nighttime hours. Nighttime enforcement of seat belt laws is encouraged because seat belt use is lower at night (2).

Additional information from CDC on preventing motor vehicle crash-related injuries is available at http://www.cdc.gov/Motorvehiclesafety. Information on state-specific seat belt use and strategies to improve it is available at http://www.cdc.gov/motorvehiclesafety/seatbelts/states.html.

## Footnotes

* Additional information is available at http://www.nhtsa.gov/Driving+Safety/Occupant+Protection.
